# *N*′-[(*E*)-(1-Methyl-1*H*-pyrrol-2-yl)methyl­idene]pyridine-4-carbohydrazide

**DOI:** 10.1107/S1600536810025341

**Published:** 2010-06-30

**Authors:** Abid Hussain, M. Nawaz Tahir, Zahid Shafiq, Muhammad Yaqub, Muhammad Mazhar

**Affiliations:** a Department of Chemistry, Bahauddin Zakariya University, Multan 60800, Pakistan; b Department of Physics, University of Sargodha, Sargodha, Pakistan; c Bahauddin Zakariya University, Department of Chemistry, Multan 60800, Pakistan

## Abstract

In the title compound, C_12_H_12_N_4_O, the pyridine and pyrrole rings are inclined at an angle of 29.22 (8)° and an intra­molecular C—H⋯N inter­action geneates an *S*(6) ring. In the crystal, mol­ecules are linked by N—H⋯N hydrogen bonds, forming (010) *C*(7) chains. The chains are cross-linked by weak C—H⋯O inter­actions, which generate *R*_2_^2^(18) ring motifs within an infinite sheet. Finally, two C—H⋯π inter­actions are present, where the C—H groups are from the pyridine ring and π is the pyrrole ring.

## Related literature

For background information on Schiff bases containing heterocyclic rings and for related structures, see: Shafiq *et al.*,(2009*a* ▶,*b* ▶); Hussain *et al.* (2010 ▶) For graph-set notation, see: Bernstein *et al.* (1995 ▶).
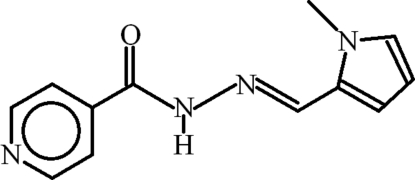


## Experimental

### 

#### Crystal data

C_12_H_12_N_4_O
*M* *_r_* = 228.26
Monoclinic, 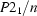

*a* = 8.2134 (3) Å
*b* = 10.6740 (4) Å
*c* = 13.1332 (4) Å
β = 96.938 (2)°
*V* = 1142.95 (7) Å^3^
*Z* = 4
Mo *K*α radiation
μ = 0.09 mm^−1^
*T* = 296 K
0.24 × 0.18 × 0.15 mm

#### Data collection

Bruker Kappa APEXII CCD diffractometer
Absorption correction: multi-scan (*SADABS*; Bruker, 2005 ▶) *T*_min_ = 0.980, *T*_max_ = 0.985
12030 measured reflections
2803 independent reflections
2023 reflections with *I* > 2σ(*I*)
*R*_int_ = 0.029

#### Refinement

*R*[*F* ^2^ > 2σ(*F* ^2^)] = 0.045
*wR*(*F* ^2^) = 0.127
*S* = 1.04
2803 reflections
155 parameters
H-atom parameters constrained
Δρ_max_ = 0.28 e Å^−3^
Δρ_min_ = −0.21 e Å^−3^

### 

Data collection: *APEX2* (Bruker, 2009 ▶); cell refinement: *SAINT* (Bruker, 2009 ▶); data reduction: *SAINT*; program(s) used to solve structure: *SHELXS97* (Sheldrick, 2008 ▶); program(s) used to refine structure: *SHELXL97* (Sheldrick, 2008 ▶); molecular graphics: *ORTEP-3 for Windows* (Farrugia, 1997 ▶) and *PLATON* (Spek, 2009 ▶); software used to prepare material for publication: *WinGX* (Farrugia, 1999 ▶) and *PLATON*.

## Supplementary Material

Crystal structure: contains datablocks global, I. DOI: 10.1107/S1600536810025341/hb5530sup1.cif

Structure factors: contains datablocks I. DOI: 10.1107/S1600536810025341/hb5530Isup2.hkl

Additional supplementary materials:  crystallographic information; 3D view; checkCIF report

## Figures and Tables

**Table 1:** Hydrogen-bond geometry (Å, °) *Cg*1 is the centroid of the C8—C11/N4 ring.

*D*—H⋯*A*	*D*—H	H⋯*A*	*D*⋯*A*	*D*—H⋯*A*
N2—H2⋯N1^i^	0.86	2.19	3.0205 (18)	163
C4—H4⋯O1^ii^	0.93	2.54	3.3821 (19)	150
C12—H12*B*⋯O1^iii^	0.96	2.55	3.450 (2)	156
C12—H12*C*⋯N3	0.96	2.36	3.025 (2)	126
C2—H2*A*⋯*Cg*1^iv^	0.93	2.83	3.3258 (16)	114
C5—H5⋯*Cg*1^v^	0.93	2.71	3.4669 (17)	139

Symmetry codes: (i) 
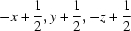
; (ii) 
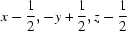
; (iii) 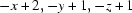; (iv) 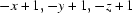; (v) 
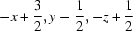
.

**Fig. 1.**
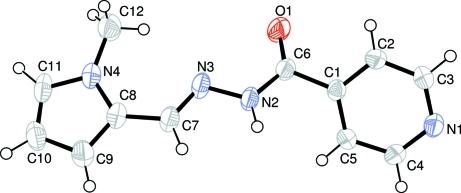
View of (I) with displacement ellipsoids drawn at the 50% probability level. H-atoms are shown by small circles of arbitrary radius.

**Fig. 2.**
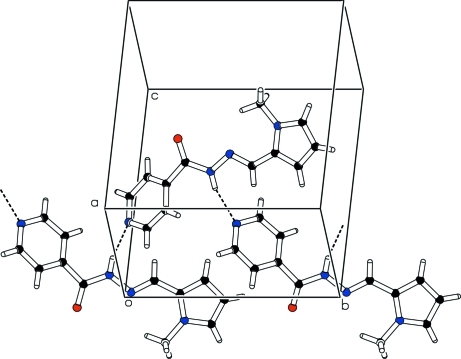
The partial packing of (I), which shows that molecules form infinite one dimensional polymeric chains extending along b axis.

**Fig. 3.**
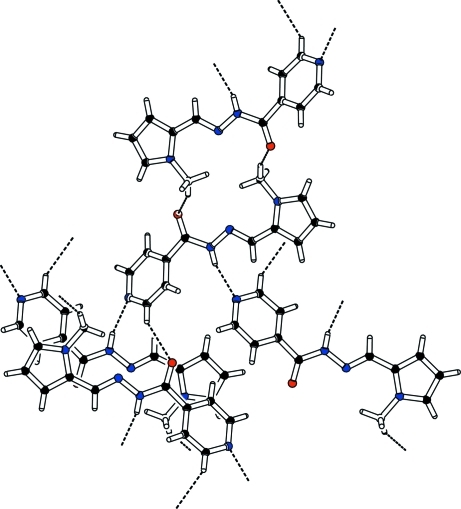
The partial packing (PLATON; Spek, 2009) which shows that molecules form R22(18) ring motifs in the infinite polymeric chains.

## supplementary crystallographic information

### Comment

We have reported crystal structures of Schiff bases with N-containing aromatic ring (Shafiq *et al.*, 2009*a*, 2009*b*), (Hussain *et al.*, 2010) and as a part of this project, we report herein the structure and synthesis of the title compound (I, Fig. 1).

In (I) the pyridine ring A (C1–C5/N1), the central moiety B (O1/C6/N2/N3/C7) and the pyrrol moiety C (C8—C11/N4/C12) are planar with r. m. s. deviations of 0.0345, 0.0285 and 0.0276 Å, respectively. The dihedral angle between A/B, A/C and B/C is 38.32 (8)°, 29.22 (8)° and 9.44 (13)°, respectively. In title molecule, there exist intra as well inter-molecular H-bondings (Table 1). The molecules form infinite one dimensional polymeric chains extending along the *b* axis (Fig. 2), if only strong H-bondings are considered. If the strong as well as weak H-bondings are considered then the molecules form two-dimensional polymeric chains with *R*_2_^2^(18) (Bernstein *et al.*, 1995) ring motifs (Fig. 3). The C—H···π interactions (Table 1) also play important role in stabilizing the molecules.

### Experimental

To a hot stirred solution of isoniazid (1.37 g, 0.01 mole) in ethanol 15 ml was added *N*-methylpyrrol-2-carboxaldehyde (1.1 ml, 0.01 mol). The resultant mixture was then heated under reflux. The reaction was monitored through TLC. After an hour, the precipitate were formed. The reaction mixture was further heated for 30 min. The resultant crude material was recrystalized in 1,4-dioxane:ethanol (1:4) to affoard red prisms of (I).

### Refinement

The H-atoms were positioned geometrically (N–H = 0.86 Å, `C–H = 0.93–0.96 Å) and refined as riding with *U*_iso_(H) = *xU*_eq_(C, N), where *x* = 1.5 for methyl and *x* = 1.2 for all other H-atoms.

### Crystal data

**Table:**

C_12_H_12_N_4_O	*F*(000) = 480
*M**_r_* = 228.26	*D*_x_ = 1.326 Mg m^−^^3^
Monoclinic, *P*2_1_/*n*	Mo *K*α radiation, λ = 0.71073 Å
Hall symbol: -P 2yn	Cell parameters from 1770 reflections
*a* = 8.2134 (3) Å	θ = 2.6–28.4°
*b* = 10.6740 (4) Å	µ = 0.09 mm^−^^1^
*c* = 13.1332 (4) Å	*T* = 296 K
β = 96.938 (2)°	Prism, red
*V* = 1142.95 (7) Å^3^	0.24 × 0.18 × 0.15 mm
*Z* = 4	

### Data collection

**Table:**

Bruker Kappa APEXII CCD diffractometer	2803 independent reflections
Radiation source: fine-focus sealed tube	2023 reflections with *I* > 2σ(*I*)
graphite	*R*_int_ = 0.029
Detector resolution: 7.50 pixels mm^-1^	θ_max_ = 28.3°, θ_min_ = 2.5°
ω scans	*h* = −10→10
Absorption correction: multi-scan (*SADABS*; Bruker, 2005)	*k* = −14→14
*T*_min_ = 0.980, *T*_max_ = 0.985	*l* = −17→17
12030 measured reflections	

### Refinement

**Table:**

Refinement on *F*^2^	Primary atom site location: structure-invariant direct methods
Least-squares matrix: full	Secondary atom site location: difference Fourier map
*R*[*F*^2^ > 2σ(*F*^2^)] = 0.045	Hydrogen site location: inferred from neighbouring sites
*wR*(*F*^2^) = 0.127	H-atom parameters constrained
*S* = 1.04	*w* = 1/[σ^2^(*F*_o_^2^) + (0.0618*P*)^2^ + 0.235*P*] where *P* = (*F*_o_^2^ + 2*F*_c_^2^)/3
2803 reflections	(Δ/σ)_max_ < 0.001
155 parameters	Δρ_max_ = 0.28 e Å^−^^3^
0 restraints	Δρ_min_ = −0.21 e Å^−^^3^

### Special details

**Table:**

Geometry. Bond distances, angles *etc*. have been calculated using the rounded fractional coordinates. All su's are estimated from the variances of the (full) variance-covariance matrix. The cell e.s.d.'s are taken into account in the estimation of distances, angles and torsion angles
Refinement. Refinement of *F*^2^ against ALL reflections. The weighted *R*-factor *wR* and goodness of fit *S* are based on *F*^2^, conventional *R*-factors *R* are based on *F*, with *F* set to zero for negative *F*^2^. The threshold expression of *F*^2^ > σ(*F*^2^) is used only for calculating *R*-factors(gt) *etc*. and is not relevant to the choice of reflections for refinement. *R*-factors based on *F*^2^ are statistically about twice as large as those based on *F*, and *R*- factors based on ALL data will be even larger.

### Fractional atomic coordinates and isotropic or equivalent isotropic displacement parameters (Å^2^)

**Table:**

	*x*	*y*	*z*	*U*_iso_*/*U*_eq_	
O1	0.68517 (16)	0.25900 (11)	0.46989 (10)	0.0596 (4)	
N1	0.25031 (16)	0.01556 (12)	0.25463 (10)	0.0424 (4)	
N2	0.55170 (15)	0.41010 (11)	0.37015 (10)	0.0388 (4)	
N3	0.65784 (16)	0.50489 (12)	0.40795 (10)	0.0417 (4)	
N4	0.85194 (16)	0.74061 (12)	0.46127 (10)	0.0411 (4)	
C1	0.46019 (16)	0.19827 (12)	0.34909 (10)	0.0315 (4)	
C2	0.40449 (19)	0.10059 (13)	0.40461 (11)	0.0374 (4)	
C3	0.30152 (19)	0.01259 (14)	0.35500 (12)	0.0414 (5)	
C4	0.3056 (2)	0.11012 (14)	0.20128 (11)	0.0421 (5)	
C5	0.40918 (19)	0.20229 (14)	0.24441 (11)	0.0378 (4)	
C6	0.57736 (18)	0.29160 (14)	0.40312 (11)	0.0371 (4)	
C7	0.62947 (19)	0.61063 (14)	0.36395 (12)	0.0416 (5)	
C8	0.72195 (19)	0.72388 (14)	0.38651 (12)	0.0404 (5)	
C9	0.6991 (2)	0.83500 (15)	0.33312 (13)	0.0497 (5)	
C10	0.8163 (2)	0.92006 (16)	0.37628 (13)	0.0539 (6)	
C11	0.9081 (2)	0.85957 (15)	0.45424 (13)	0.0496 (6)	
C12	0.9127 (2)	0.65484 (17)	0.54194 (14)	0.0574 (6)	
H2	0.46924	0.42717	0.32551	0.0466*	
H2A	0.43635	0.09445	0.47482	0.0449*	
H3	0.26551	−0.05268	0.39352	0.0497*	
H4	0.27213	0.11375	0.13112	0.0505*	
H5	0.44438	0.26614	0.20412	0.0454*	
H7	0.54104	0.61488	0.31268	0.0500*	
H9	0.61937	0.85044	0.27801	0.0597*	
H10	0.82924	1.00246	0.35569	0.0646*	
H11	0.99575	0.89429	0.49628	0.0595*	
H12A	0.86108	0.67234	0.60217	0.0860*	
H12B	1.02928	0.66469	0.55737	0.0860*	
H12C	0.88828	0.57043	0.51995	0.0860*	

### Atomic displacement parameters (Å^2^)

**Table:**

	*U*^11^	*U*^22^	*U*^33^	*U*^12^	*U*^13^	*U*^23^
O1	0.0566 (8)	0.0471 (7)	0.0666 (8)	−0.0071 (6)	−0.0275 (6)	0.0054 (6)
N1	0.0438 (8)	0.0334 (7)	0.0475 (7)	−0.0070 (6)	−0.0048 (5)	−0.0019 (5)
N2	0.0359 (7)	0.0296 (6)	0.0480 (7)	−0.0057 (5)	−0.0070 (5)	−0.0040 (5)
N3	0.0389 (7)	0.0336 (7)	0.0511 (7)	−0.0081 (5)	−0.0010 (5)	−0.0087 (5)
N4	0.0427 (7)	0.0335 (7)	0.0474 (7)	−0.0083 (5)	0.0062 (6)	−0.0074 (5)
C1	0.0297 (7)	0.0266 (7)	0.0374 (7)	0.0006 (5)	0.0003 (5)	−0.0018 (5)
C2	0.0437 (8)	0.0331 (8)	0.0340 (7)	−0.0001 (6)	−0.0012 (6)	0.0025 (6)
C3	0.0458 (9)	0.0331 (8)	0.0449 (8)	−0.0071 (7)	0.0035 (6)	0.0052 (6)
C4	0.0509 (9)	0.0385 (8)	0.0341 (7)	−0.0045 (7)	−0.0061 (6)	−0.0011 (6)
C5	0.0453 (9)	0.0318 (7)	0.0359 (7)	−0.0047 (6)	0.0030 (6)	0.0032 (6)
C6	0.0348 (8)	0.0343 (8)	0.0406 (7)	−0.0037 (6)	−0.0022 (6)	−0.0022 (6)
C7	0.0380 (8)	0.0344 (8)	0.0509 (9)	−0.0047 (7)	−0.0012 (7)	−0.0074 (7)
C8	0.0396 (8)	0.0351 (8)	0.0470 (8)	−0.0051 (6)	0.0069 (7)	−0.0087 (6)
C9	0.0580 (10)	0.0381 (9)	0.0529 (9)	−0.0047 (8)	0.0064 (8)	−0.0029 (7)
C10	0.0729 (12)	0.0331 (8)	0.0580 (10)	−0.0118 (8)	0.0178 (9)	−0.0040 (7)
C11	0.0560 (10)	0.0380 (9)	0.0570 (10)	−0.0198 (8)	0.0162 (8)	−0.0155 (8)
C12	0.0538 (11)	0.0461 (10)	0.0680 (11)	−0.0058 (8)	−0.0098 (8)	0.0025 (9)

### Geometric parameters (Å, °)

**Table:**

O1—C6	1.219 (2)	C7—C8	1.439 (2)
N1—C3	1.335 (2)	C8—C9	1.379 (2)
N1—C4	1.339 (2)	C9—C10	1.393 (2)
N2—N3	1.3877 (18)	C10—C11	1.359 (2)
N2—C6	1.3453 (19)	C2—H2A	0.9300
N3—C7	1.277 (2)	C3—H3	0.9300
N4—C8	1.372 (2)	C4—H4	0.9300
N4—C11	1.358 (2)	C5—H5	0.9300
N4—C12	1.443 (2)	C7—H7	0.9300
N2—H2	0.8600	C9—H9	0.9300
C1—C2	1.3815 (19)	C10—H10	0.9300
C1—C5	1.3886 (19)	C11—H11	0.9300
C1—C6	1.502 (2)	C12—H12A	0.9600
C2—C3	1.374 (2)	C12—H12B	0.9600
C4—C5	1.377 (2)	C12—H12C	0.9600
			
C3—N1—C4	116.64 (13)	N4—C11—C10	109.47 (15)
N3—N2—C6	120.21 (12)	C1—C2—H2A	120.00
N2—N3—C7	114.22 (13)	C3—C2—H2A	120.00
C8—N4—C11	108.33 (13)	N1—C3—H3	118.00
C8—N4—C12	127.81 (13)	C2—C3—H3	118.00
C11—N4—C12	123.58 (14)	N1—C4—H4	118.00
C6—N2—H2	120.00	C5—C4—H4	118.00
N3—N2—H2	120.00	C1—C5—H5	121.00
C2—C1—C6	119.03 (12)	C4—C5—H5	121.00
C2—C1—C5	117.80 (13)	N3—C7—H7	117.00
C5—C1—C6	123.13 (12)	C8—C7—H7	117.00
C1—C2—C3	119.32 (13)	C8—C9—H9	126.00
N1—C3—C2	123.67 (14)	C10—C9—H9	126.00
N1—C4—C5	123.72 (14)	C9—C10—H10	127.00
C1—C5—C4	118.86 (13)	C11—C10—H10	127.00
N2—C6—C1	113.89 (12)	N4—C11—H11	125.00
O1—C6—N2	124.87 (14)	C10—C11—H11	125.00
O1—C6—C1	121.24 (13)	N4—C12—H12A	109.00
N3—C7—C8	125.91 (15)	N4—C12—H12B	109.00
N4—C8—C9	107.35 (13)	N4—C12—H12C	109.00
C7—C8—C9	125.57 (15)	H12A—C12—H12B	109.00
N4—C8—C7	127.04 (14)	H12A—C12—H12C	109.00
C8—C9—C10	107.99 (15)	H12B—C12—H12C	109.00
C9—C10—C11	106.87 (15)		
			
C4—N1—C3—C2	0.6 (2)	C2—C1—C5—C4	0.4 (2)
C3—N1—C4—C5	−0.5 (2)	C6—C1—C5—C4	177.90 (14)
C6—N2—N3—C7	173.81 (14)	C2—C1—C6—O1	38.1 (2)
N3—N2—C6—O1	4.1 (2)	C2—C1—C6—N2	−142.47 (14)
N3—N2—C6—C1	−175.38 (12)	C5—C1—C6—O1	−139.38 (16)
N2—N3—C7—C8	−178.82 (14)	C5—C1—C6—N2	40.08 (19)
C11—N4—C8—C7	177.64 (15)	C1—C2—C3—N1	−0.2 (2)
C11—N4—C8—C9	0.04 (17)	N1—C4—C5—C1	0.0 (2)
C12—N4—C8—C7	−8.4 (3)	N3—C7—C8—N4	−2.9 (3)
C12—N4—C8—C9	174.00 (15)	N3—C7—C8—C9	174.33 (16)
C8—N4—C11—C10	0.12 (19)	N4—C8—C9—C10	−0.18 (18)
C12—N4—C11—C10	−174.15 (15)	C7—C8—C9—C10	−177.82 (15)
C5—C1—C2—C3	−0.3 (2)	C8—C9—C10—C11	0.25 (19)
C6—C1—C2—C3	−177.89 (14)	C9—C10—C11—N4	−0.23 (19)

### Hydrogen-bond geometry (Å, °)

**Table:**

Cg1 is the centroid of the C8—C11/N4 ring.

**Table:**

*D*—H···*A*	*D*—H	H···*A*	*D*···*A*	*D*—H···*A*
N2—H2···N1^i^	0.86	2.19	3.0205 (18)	163
C4—H4···O1^ii^	0.93	2.54	3.3821 (19)	150
C12—H12B···O1^iii^	0.96	2.55	3.450 (2)	156
C12—H12C···N3	0.96	2.36	3.025 (2)	126
C2—H2A···Cg1^iv^	0.93	2.83	3.3258 (16)	114
C5—H5···Cg1^v^	0.93	2.71	3.4669 (17)	139

Symmetry codes: (i) −*x*+1/2, *y*+1/2, −*z*+1/2; (ii) *x*−1/2, −*y*+1/2, *z*−1/2; (iii) −*x*+2, −*y*+1, −*z*+1; (iv) −*x*+1, −*y*+1, −*z*+1; (v) −*x*+3/2, *y*−1/2, −*z*+1/2.
