# *N*′-[(*E*)-(1-Methyl-1*H*-pyrrol-2-yl)methyl­idene]pyridine-4-carbohydrazide. Corrigendum

**DOI:** 10.1107/S1600536810031090

**Published:** 2010-08-11

**Authors:** Abid Hussain, M. Nawaz Tahir, Zahid Shafiq, Muhammad Yaqub, Mazhar Hussain

**Affiliations:** a Department of Chemistry, Bahauddin Zakariya University, Multan 60800, Pakistan; b Department of Physics, University of Sargodha, Sargodha, Pakistan

## Abstract

The name of one of the authors in the paper by Hussain *et al.* [*Acta Cryst.* (2010), E**66**, o1881] is corrected.

## Abstract 2

Corrigendum to *Acta Cryst.* (2010), E**66**, o1881.

In the paper by Hussain *et al.* (2010) ▶, the last author is incorrectly given as ‘Muhammad Mazhar’. The correct name of the last author should be ‘Mazhar Hussain’ as above.
